# Transposase mapping identifies the genomic targets of BAP1 in uveal melanoma

**DOI:** 10.1186/s12920-018-0424-0

**Published:** 2018-11-06

**Authors:** Matthew Yen, Zongtai Qi, Xuhua Chen, John A. Cooper, Robi D. Mitra, Michael D. Onken

**Affiliations:** 1 Department of Biochemistry and Molecular Biophysics, Washington University School of Medicine, 660 S. Euclid Ave., St. Louis, MO 63110 USA; 2 Department of Genetics and Center for Genome Sciences and Systems Biology, Washington University School of Medicine, 660 S. Euclid Ave., St. Louis, MO 63110 USA

**Keywords:** Genomic mapping, Transcription, BAP1, Uveal melanoma

## Abstract

**Background:**

BAP1 is a histone deubiquitinase that acts as a tumor and metastasis suppressor associated with disease progression in human cancer. We have used the “Calling Card System” of transposase-directed transposon insertion mapping to identify the genomic targets of BAP1 in uveal melanoma (UM). This system was developed to identify the genomic loci visited by transcription factors that bind directly to DNA; our study is the first use of the system with a chromatin-remodeling factor that binds to histones but does not interact directly with DNA.

**Methods:**

The transposase *piggyBac* (PBase) was fused to BAP1 and expressed in OCM-1A UM cells. The insertion of transposons near BAP1 binding sites in UM cells were identified by genomic sequencing. We also examined RNA expression in the same OCM-1A UM cells after BAP1 depletion to identify BAP1 binding sites associated with BAP1-responsive genes. Sets of significant genes were analyzed for common pathways, transcription factor binding sites, and ability to identify molecular tumor classes.

**Results:**

We found a strong correlation between multiple calling-card transposon insertions targeted by BAP1-PBase and BAP1-responsive expression of adjacent genes. BAP1-bound genomic loci showed narrow distributions of insertions and were near transcription start sites, consistent with recruitment of BAP1 to these sites by specific DNA-binding proteins. Sequence consensus analysis of BAP1-bound sites showed enrichment of motifs specific for YY1, NRF1 and Ets transcription factors, which have been shown to interact with BAP1 in other cell types. Further, a subset of the BAP1 genomic target genes was able to discriminate aggressive tumors in published gene expression data from primary UM tumors.

**Conclusions:**

The calling card methodology works equally well for chromatin regulatory factors that do not interact directly with DNA as for transcription factors. This technique has generated a new and expanded list of BAP1 targets in UM that provides important insight into metastasis pathways and identifies novel potential therapeutic targets.

**Electronic supplementary material:**

The online version of this article (10.1186/s12920-018-0424-0) contains supplementary material, which is available to authorized users.

## Background

BAP1 is a histone deubiquitinase that remodels chromatin to regulate gene expression. The BAP1 polypeptide is the catalytic subunit of the polycomb-repressive deubiquitinase complex, which requires either ASXL1 or ASXL2 [1], and which can include HCFC1, OGT and other factors [2]. This complex, a component of the polycomb pathway, removes mono-ubiquitin from histone H2A [1]. The HCFC1 subunit is a transcriptional co-activator that can bind transcription factors such as E2F, YY1, and Ets-related transcription factors [3, 4]; however, its role in targeting BAP1 to chromatin has not been fully elucidated.

Melanomas arising from the pigmented layers (uvea) of the eye are highly aggressive cancers: almost half of patients with uveal melanoma (UM) die from metastatic disease, even after the primary tumor is completely removed by surgical excision of the eye [5], because we are unable to prevent or treat metastatic spread of the cancer [5]. UMs can be divided into two classes by molecular and genetic analysis of the tumor. Class 1 UMs have a favorable prognosis; the cancers are low-grade, indolent, and rarely metastasize. Class 2 UMs have a dismal prognosis; they are high-grade, aggressive and nearly always metastasize [6]. Over 95% of class 2 UMs show complete loss of expression of BAP1 protein [7], with inactivating somatic mutations in the *BAP1* gene in 80% of these tumors [8]. Loss of BAP1 causes UM cells to assume a rounded, epithelioid morphology, to deposit distinctive extracellular matrix materials, and to grow well under clonogenic conditions [9, 10], and BAP1-depleted UM cells display increased diapedesis through endothelial monolayers in a cell-culture model of transendothelial migration [11], which may reflect their ability to metastasize. BAP1 mutations have been found in other aggressive cancers, including skin-derived melanomas, mesotheliomas, and renal cell carcinomas [12–17], suggesting a general role for BAP1 as a suppressor of metastasis in cancer.

Transposon integration by targeted transposases has been used to identify genomic regions in several contexts [18]. The coordinates and numbers of insertions of transposons reflect the locations where the factor binds and the proportion of time the factor is bound to the locus. The “calling card” methodology fuses a piggyBac transposase to a protein of interest [19], and uses multiple bar-coded transposon donor plasmids to improve the spatial and temporal demarcation of integration sites [20]. Here, we modified the technique, originally developed for DNA-binding transcription factors, to detect the interactions of chromatin with BAP1 complex, which does not bind directly to DNA. Our results provide novel insights into the biology of BAP1 in cancer tumor suppression and metastasis.

## Methods

### Cell culture and reagents

The coding region of human BAP1 cDNA (NM_004656.2) from pReceiver-M12 BAP1 (GeneCopoeia, Rockville, MD) was fused to cDNA encoding the hyperactive piggyBac transposase derived from a pCMV-hyPBase plasmid, generously provided by Dr. Allan Bradley [21]. Both N-terminal and C-terminal fusions were prepared, using Gibson assembly [22]. For BAP1-PBase, BAP1 was placed at the 5′ end of hyPBase with an 18-aa linker, KLGGGAPAVGGGPKAADK. For PBase-BAP1, BAP1 was placed at the 3′ end of hyPBase, with the same linker. Plasmid clones were maintained and expanded in DH5-α cells in carbenicillin, and the identities of plasmids were confirmed by DNA sequencing of all regions that underwent PCR amplification (Genewiz; South Plainfield, NJ). Forty uniquely bar-coded piggyBac transposon plasmids [20] were used as donors for the calling card protocol. The 40 plasmids were divided into four sets of 10 donors per experiment.

OCM-1A cells were originally derived by Dr. June Kan-Mitchell [23]. Cells were cultured in growth medium: RPMI 1640 with 10% FBS and penicillin/streptomycin (Gibco; Carlsbad, CA) at 37 °C in 5% CO_2_. Cells were transfected using TransIT-LT1 transfection reagent (Mirus; Madison, WI) according to manufacturer’s instructions. After 24 h, the medium was replaced with growth medium containing 1.4 μg/mL puromycin. Cells were maintained under selection for 2 weeks, at which point large visible colonies were formed. Colonies were harvested by trypsinization and centrifugation, and cell pellets were stored at − 80 °C.

### Preparation of genomic DNA

The following procedure was adapted and modified from [19]. Genomic DNA was isolated from cell pellets using a High MW Cell DNA Isolation Kit (EZ Bioresearch, St. Louis, MO) according to manufacturer instructions. For each sample, three independent digests were performed with Taq1, Msp1, and CviQ (New England Biolabs, Ipswich, MA) using 20 μg of genomic DNA and following manufacturer protocols. Each separate digested genomic DNA sample was purified using a Qiaquick PCR Purification Kit (Qiagen), self-ligated with T4 Ligase (New England Biolabs) at 15 °C for 18–24 h, and purified with Amicon Ultra-0.5 mL Centrifugal 30 k filters (Millipore; Billerica, MA) with 5-min spins (instead of the standard protocol of 10–30 min). The circularized genomic material from the three independent digests were pooled for each experimental sample and inverse PCR was performed using piggyBac transposon-specific primers. DNA fragments from 200 to 1000 bp were isolated and yields were quantified by UV absorbance. Next-gen sequencing was performed on the MiSeq 2 × 250 (Illumina) by the Center for Genomic Sciences and Systems Biology at Washington University in St. Louis, using the transposon-specific sequencing primer.

### Calling card data analysis

Raw reads were mapped back to the human genome (hg19) with Bowtie2. Significant Calling Cards peaks were called with a modified version of the previously described algorithm [19]. Briefly, transposon insertions were clustered into peaks with a maximum distance of 5 kb between insertions. Significant peaks were identified using the Poisson distribution to test for enrichment over the background (unfused transposase) Calling Card data. The expected number of hops per TTAA was locally estimated from the background Calling Card data by considering regions centered directly under the Calling Card peak, 1 kb from the Calling Card peak, or 5 kb from the Calling Card peak, and taking the maximum of the three estimated parameters. Peaks were annotated with nearby gene information using bedtools.

### Lentiviral shRNA knockdown of BAP1

The following was modified from Mooren et al. [24]. Lentiviral pLKO.1 shRNA plasmid targeting *BAP1* (NM_004656.2-2658s1c1) was designed by the RNAi consortium (TRC) and obtained from the Children’s Discovery Institute / Genome Sequencing Center at Washington University. Lentiviral shRNA expression plasmids were cotransfected into HEK293T cells with the plasmids pCMV-dR8.2 dvpr and pCMV-VSV-G. After 72 h, viral particles were harvested. OCM-1A cells were infected with lentivirus in growth medium with 8 μg/mL protamine sulfate. After 24 h, medium was changed to fresh growth medium containing 1.4 μg/mL puromycin. OCM-1A cells were harvested for analysis of protein and RNA 6 days post infection.

### RNA sequencing and analysis

RNA was isolated using the Qiagen RNeasy Mini Kit according to manufacturer instructions, including the optional DNase I step. Second-strand cDNA synthesis was performed using SuperScript IV (ThermoFisher Scientific; Waltham, MA) according to manufacturer instructions. Next-gen RNA sequencing was performed on the Illumina HiSeq2500 1 × 50 by the Genome Technology Access Center (Washington University). Three biological replicates for *BAP1* and control (GFP) knockdowns were sequenced. RNA-Seq analysis was performed by the Genomic Technology Access Center at Washington University. Reads were aligned to the Ensembl release 76 top-level assembly with STAR version 2.0.4b. Gene counts were derived from the number of uniquely aligned unambiguous reads by Subread:featureCount version 1.4.5. Transcript counts were produced by Sailfish version 0.6.3. Sequencing performance was assessed for total number of aligned reads, total number of uniquely aligned reads, genes and transcripts detected, ribosomal fraction known junction saturation and read distribution over known gene models with RSeQC version 2.3.

Gene-level and transcript counts were imported into the R/Bioconductor package. EdgeR and TMM normalization size factors were calculated to adjust for samples with differences in library size. Genes or transcripts not expressed in any sample were excluded from further analysis. The TMM size factors and the matrix of counts were imported into R/Bioconductor package Limma. Weighted likelihoods based on the observed mean-variance relationship of every gene/transcript and sample were calculated for all samples with the voomWithQualityWeights function. Generalized linear models were then created to test for gene/transcript level differential expression. Differentially expressed genes and transcripts were then filtered for FDR adjusted *p*-values less than or equal to 0.01. Gene ontology (GO) and pathway analyses was performed using Gene Set Enrichment Analysis (GSEA) [25]. GO groups were assembled by merging the lists of genes from related GO terms that were significantly enriched in the signature gene set. GSEA was also used to identify published molecular signatures (curated by the Broad Institute: http://broadinstitute.org/GSEA) that showed significant overlap (*p* < 0.001) with response to BAP1 depletion.

### DNA motif analysis

BAP1-PBase calling card peaks with five or more insertions were used for Hypergeometric Optimization of Motif EnRichment (HOMER) analysis (available at: http://homer.ucsd.edu). Peaks were analyzed separately depending on whether they were associated with genes showing significant change (*p* < 0.01) in expression after *BAP1* depletion, and whether the associated gene increased or decreased expression upon Bap1 depletion. HOMER was used to identify enriched motifs in genomic regions (findMotifsGenome.pl) using the given size of each peak.

## Results

### The calling card system identifies genomic regions bound by BAP1

**Fig. 1:**
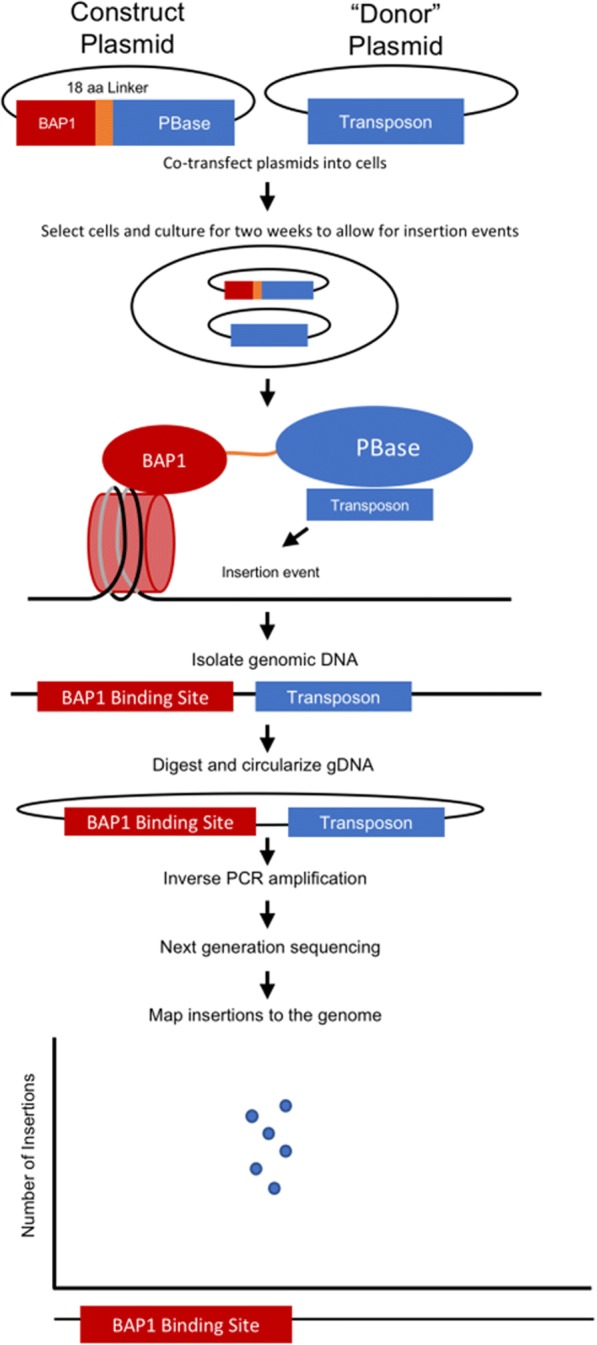
Schematic representation of experimental approach. The calling card system was used to identify regions of genomic interactions by BAP1. Plasmids were constructed to express BAP1 fused with the piggyBac transposase (PBase) via an 18-aa linker. After plasmid transfection into UM cells, expressed BAP1-PBase fusion proteins interact with nucleosomes containing ubiquitylated histone H2A via BAP1, allowing PBase to insert a barcoded transposon from a co-transfected donor plasmid into the adjacent genomic DNA. The genome is fragmented, and barcoded transposon primers are used to amplify the genomic insertion sites for each transposon. Next-gen sequencing is performed to identify the genomic insertion sites, which are mapped back to the hg38 human genome assemble

**Fig. 2:**
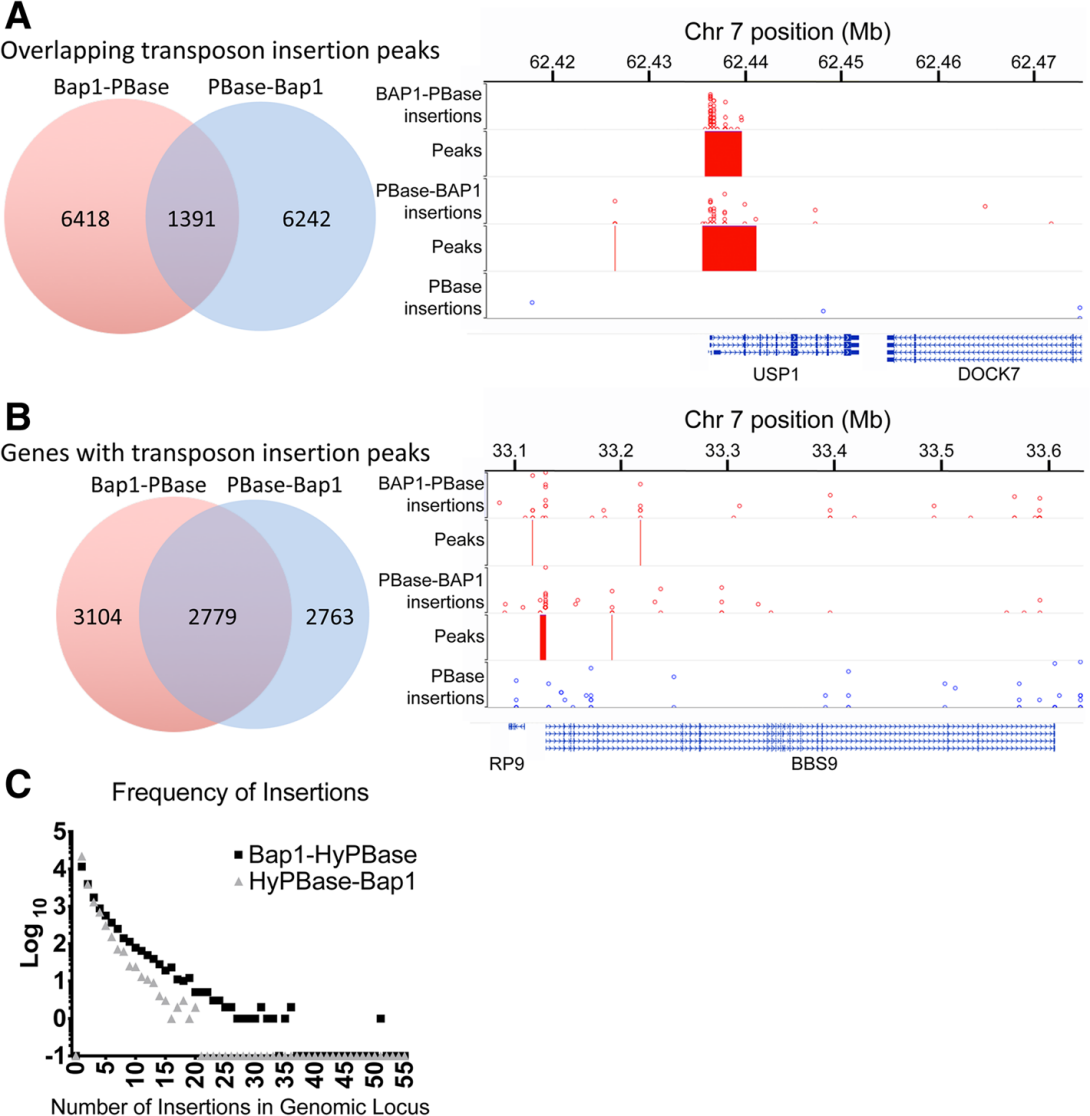
BAP1 N-terminal and C-terminal PBase fusions identified overlapping sets of target genes. **a** Genomic peak regions of transposon insertions were identified for N-terminal (BAP1-PBase) and C-terminal (PBase-BAP1) fusion constructs. Left: Venn diagram showing modest overlap of called peaks between the two constructs. Right: representative genomic region illustrating overlapping peaks. **b** Genes containing or adjacent to transposon insertion peaks were identified and compared between the two fusion constructs. Left: Venn diagram showing overlap of targeted genes. Right: representative genomic region showing a gene with non-overlapping insertion peaks. **c** Differences in the insertion rates between the two fusion constructs. The number of genomic loci (Y-axis) is plotted against the numbers of insertions per locus (X-axis). A large proportion of calling card insertions were unclustered background represented by genomic loci with 5 or fewer insertions within 10 kb. PBase-BAP1 produced proportionally fewer genomic loci with multiple insertions than did BAP1-PBase

We fused the piggyBac transposase (PBase) to BAP1 with a flexible 18-aa linker at either end of BAP1 (Fig. 1). Each fusion construct was cotransfected with PB donor plasmids into OCM-1A UM cells; unfused PBase served as a negative control. Next-gen sequencing of genomic DNA revealed specific clusters of genomic reads associated with BAP1-PBase fusion constructs and not present in control samples. The BAP1-PBase and PBase-BAP1 fusion constructs produced 199,209 and 179,244 genomic transposon insertions, respectively, and these insertion sites clustered into 7,810 (see Additional file 1) and 7,634 (see Additional file 2) genomic peaks. Peaks were called by accounting for background hops (i.e. a call required enrichment of BAP1 hops over background). Only a small subset of genomic peaks showed directly overlapping insertion peaks with both fusion constructs (Fig. 2a). For each construct, many genes were found to contained multiple, non-overlapping peaks. We clustered the genomic peaks based on closest gene (5,883 for BAP1-PBase and 5,542 for PBase-BAP1), and the targeted genes showed substantial overlap (47% for BAP1-PBase and 50% for PBase-BAP1; Fig. 2b). The BAP1-PBase fusion generated more high-number insertion sites than did the PBase-BAP1 fusion, with higher maximum numbers of transposons per locus (86 for BAP1-PBase vs. 58 for PBase-BAP1).

### BAP1 interacts with chromatin near transcription start sites

**Fig. 3:**
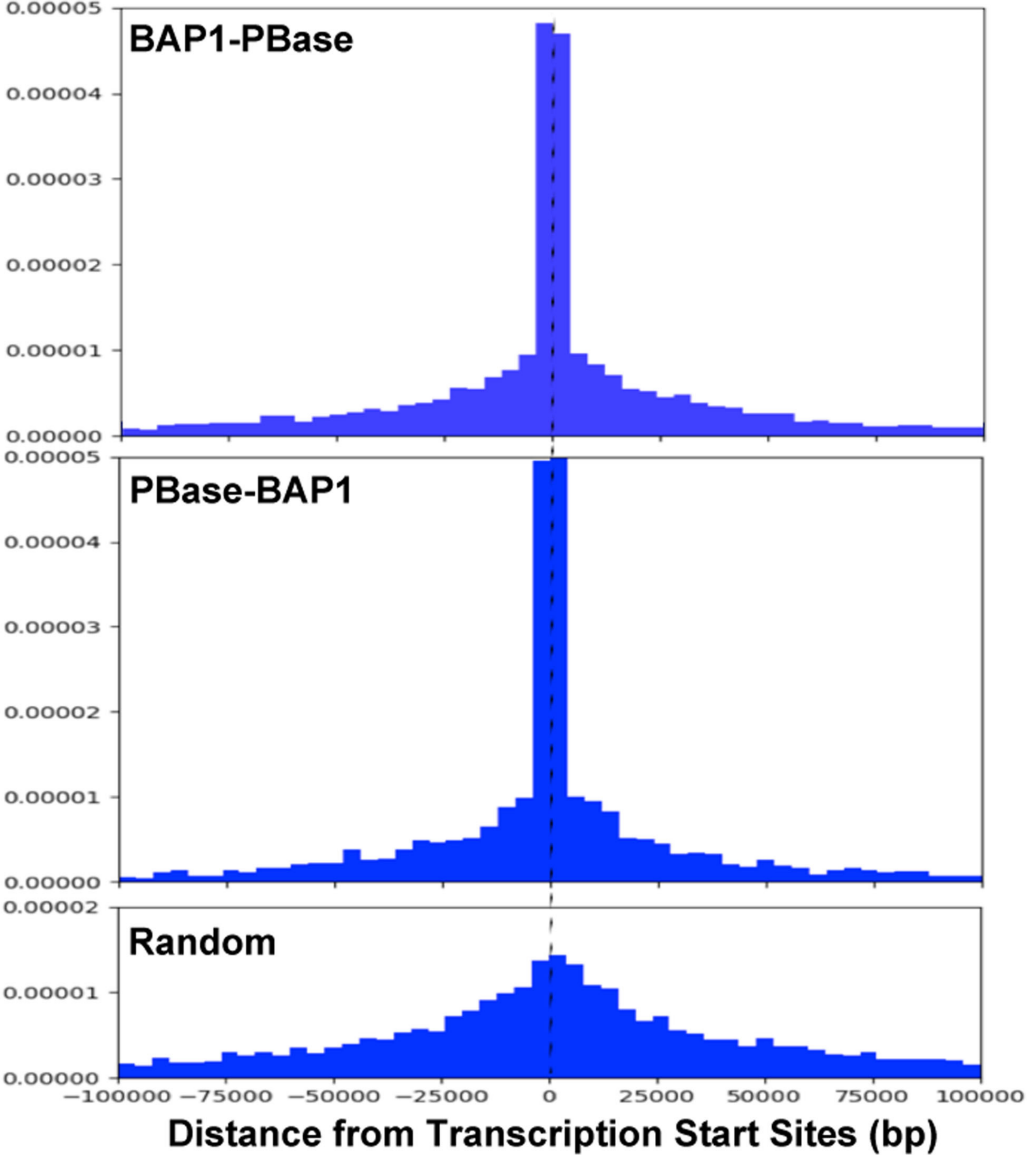
BAP1-directed transposon insertions cluster at narrow peaks near transcription start sites. Histograms showing the distance from each BAP1-PBase or PBase-BAP1 peak to the nearest transcription start site (top and middle panels), or the distance from randomly selected position of the genome to the nearest transcription start site (bottom panel). Both fusion constructs produced significantly more insertions within 5 kb of transcription start sites (*p* < 0.001)

Many genomic peaks with high numbers of insertions were narrow and centered close to transcription start sites. The distances of each BAP1-PBase or PBase-BAP1 genomic peak to the nearest transcription start site were determined and plotted as histograms (Fig. 3). As a control, we analyzed distances from transcription start sites for randomly selected regions of the genome (Fig. 3). Mann-Whitney *U* tests revealed that BAP1-PBase and PBase-BAP1 peaks were both significantly closer to transcription start sites (*p* < 0.0001 for both). One concern was that PBase alone might be predisposed to target transposons to open chromatin and thus near active transcription start sites. Comparing BAP1-fusion insertions with all background insertions, we found distances to transcription start sites as highly enriched for both fusions (p < 0.0001, Mann-Whitney *U* tests). Thus, BAP1 binds specifically near transcription start sites.

### BAP1 genomic targets show BAP1-dependent gene expression

**Fig. 4:**
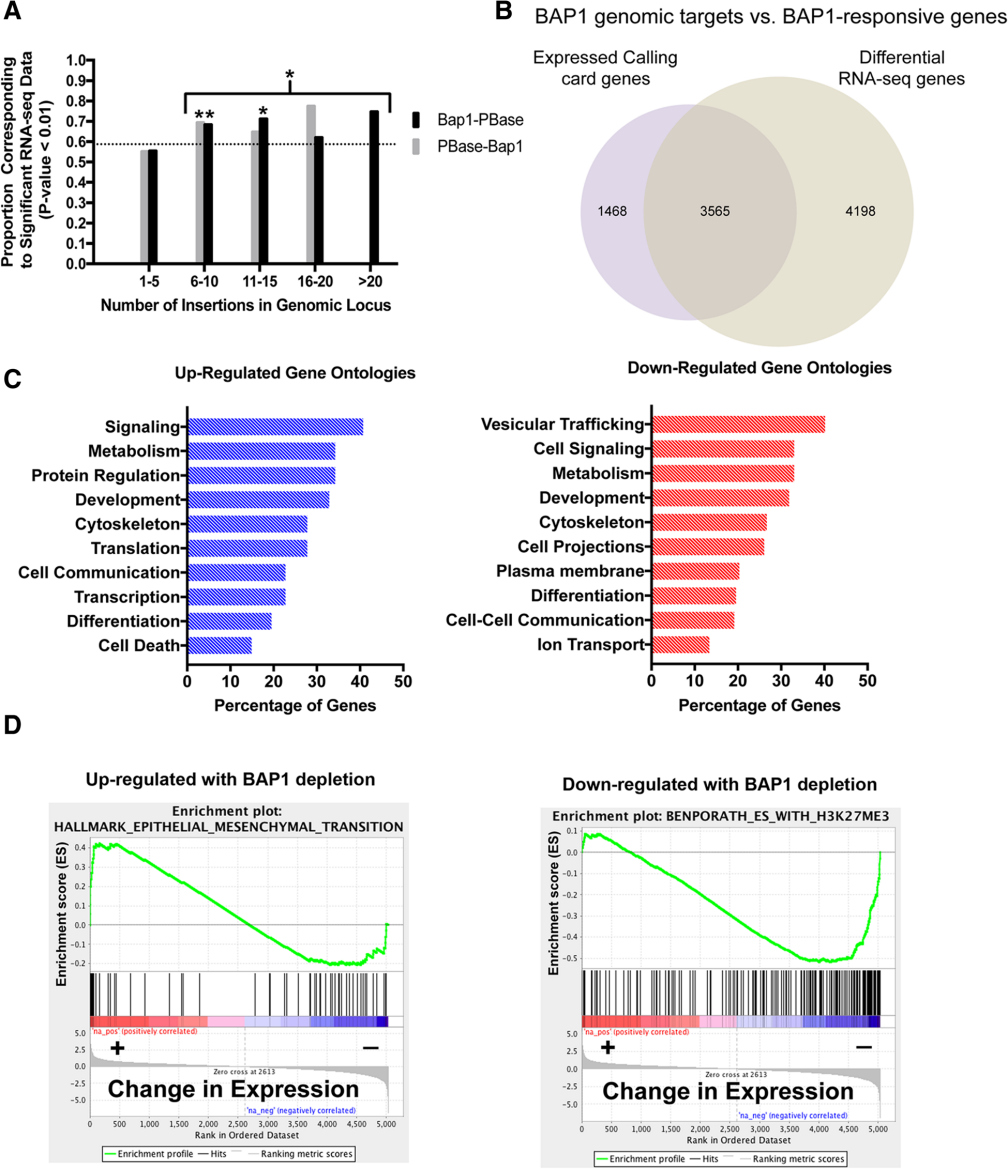
Loci with calling card insertions are associated with BAP1-regulated genes. **a** Correlation of gene expression with genomic loci calling-card insertions. For genomic loci with > 5 insertions, a statistically significant fraction show changes in expression, based on RNA-Seq, following loss of BAP1. **b** Venn diagram showing the overlap between BAP1 target genes > 5 calling-card insertion peaks and genes for which expression changed significantly (*p* < 0.01) following BAP1 depletion, as assessed by RNA-Seq. **c** Gene ontology pathway analysis was performed on BAP1 target genes divided into sets that were up-regulated or down-regulated in response to BAP1 depletion. Only significant pathways are shown (*p* < 0.01). **d** Two highly significant (*p* < 0.001) molecular signatures were identified by Gene Set Enrichment Analysis of BAP1 calling-card target genes associated with gene expression response to BAP1 depletion: the Epithelial-to-Mesenchymal Transition (EMT) signature was associated with up-regulated genes; and the polycomb-mediated lysine27-trimethylation of histone H3 in Embryonic Stem Cells was associated with down-regulated genes

To ascertain whether genes targeted by BAP1 in the calling card analysis were functionally regulated by BAP1, we performed RNA-Seq on OCM-1A cells depleted of BAP1 by lentiviral shRNAs targeting BAP1. GFP-targeting shRNAs served as a negative control [10]. BAP1 protein levels decreased by 68–74%, compared to GFP controls, by immunoblot (see Additional file 3). BAP1 transcript levels decreased similarly, to 75%, compared to controls based on RNA-seq (see Additional file 4). RNA-Seq results revealed a significant response for 70% (3,565 / 5,033) of BAP1-targeted genes with > 5 insertions. Among the significant gene targets, 22% (784 / 3,565) exhibited greater than two-fold changes in expression in response to BAP1 depletion (Fig. 4a-b and (Additional file 5)). Genes with five or fewer insertions did not show a significant association with expression changes (Fisher’s exact test, *p* > 0.01; Fig. 4a). This level of concordance between genomic insertion and expression change is remarkable. By comparison, a study of multiple transcription factors found that less than 15% of transcription factor-bound target genes were perturbed upon knockdown of the transcription factor [26].

We performed pathway and gene ontology analyses on the 784 genes identified by the combination of > 2-fold BAP1-sensitive RNA expression changes and calling-card transposon targeting. The top three pathways enriched for the 244 up-regulated targets were the HIF1α, β3-integrin signaling, and p53 signaling pathways (see Additional file 6). The top three pathways enriched for the 540 down-regulated targets were the Gαi (G-protein), PDGF-Rβ signaling, and the EGFR signaling pathways (see Additional file 6). Gene ontology analysis identified significant differential regulation of ontologies associated with development and differentiation, and with the cytoskeleton (Fig. 4c). BAP1-depletion was associated with up-regulation of genes involved in transcription, translation, and protein regulation (Fig. 4c), and down-regulation of genes involved in vesicular transport, membrane formation, and cell projections (Fig. 4c). Two highly significant (*p* < 0.001) molecular signatures were identified by Gene Set Enrichment Analysis of BAP1 calling-card target genes associated with gene expression response to BAP1 depletion (see Additional file 7): an Epithelial-to-Mesenchymal Transition (EMT) signature was associated with up-regulated genes; and a polycomb-mediated lysine27-trimethylation of histone H3 in Embryonic Stem Cells was associated with down-regulated genes (Fig. 4d). These analyses suggest that, in UM cells, BAP1 coordinates several downstream pathways through its genomic targets that are important to metastatic spread and survival by regulating differentiation, lineage specificity and stemness.

### BAP1 is recruited to specific DNA-binding motifs

Our data suggested recruitment of BAP1 to specific genes by DNA-binding proteins, as opposed to random scanning for monoubiquitylated histones. Genomic loci associated with genes that were up-regulated versus down-regulated in response to BAP1 depletion were analyzed separately (see Additional file 8). HOMER analysis is designed for use with large data sets (~ 10,000 peaks), so de novo motif analysis could not be performed due to the small number of peaks in each data set (approximately 500–600) being analyzed. Enrichment was found for a number of motifs bound by transcription factors that have been previously identified to form complexes with BAP1 [4], including NRF1, Ets factors, and YY1. Surprisingly, the up- and down-regulated gene sets were both enriched for NRF1 and YY1 suggesting that other transcription factors on the same promoters are determining direction and magnitude of change in gene expression. On the other hand, only up-regulated genes were enriched for Ets factor binding sites, suggesting the existence of a BAP1-containing co-activator complex specifically associated with Ets-factors.

### BAP1 calling card targets are linked to the metastatic signature

**Fig. 5:**
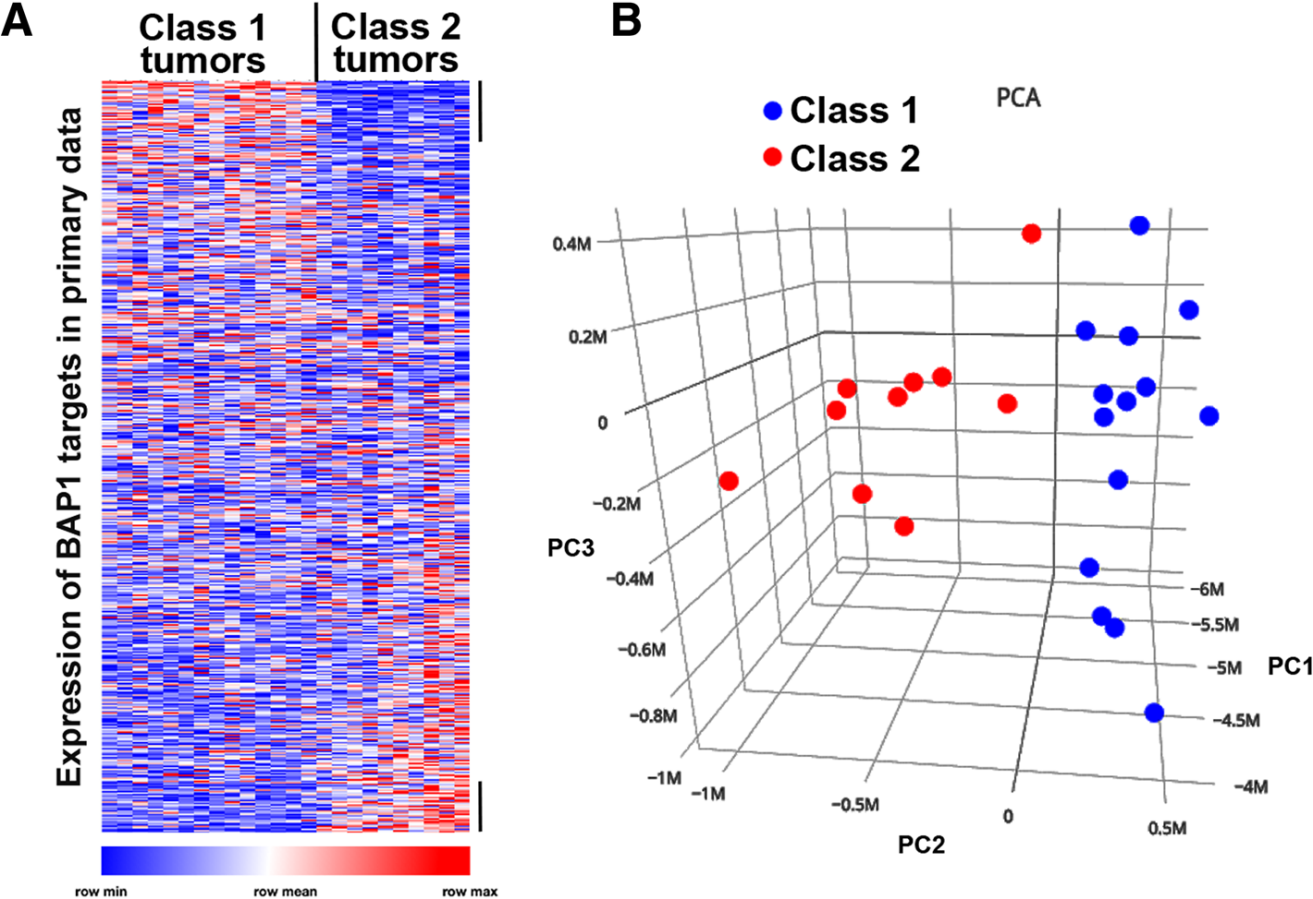
BAP1 calling-card target genes distinguish molecular class in primary UM tumors. Gene expression values from published microarray data of primary UM tumors [6] were analyzed for genes identified in this study as having BAP1-targeted insertions and significant gene expression response to BAP1 depletion. **a** Heatmap showing relative gene expression (blue to red) of BAP1 targets in primary UM tumors. Each column is one human tumor. Each row is one gene. Genes were clustered by comparative marker selection based on tumor class (see Additional file 9). Maximum and minimum values were normalized for each row and do not represent global maxima or minima. Black bars indicate genes with significant differential expression (*p* < 0.01). **b** Unsupervised principal component analysis of published tumor data using only BAP1 calling card targets shows a portion of genes (principal component 2: PC2) that distinguish class 1 (blue circles) from class 2 (red circles) tumors

UM tumors are classified clinically by gene expression profiling of primary tumor samples [6, 27, 28]. We cross-referenced our list of 784 highly significant BAP1 genomic target genes to the list of genes from primary tumor expression analysis [6]. We used comparative marker selection to cluster the genes based on tumor class to determine whether BAP1-responsive genomic target genes correlated with the phenotypic distinction between class 1 and class 2 tumors, (Fig. 5a) (see Additional file 9). We found that a subset of BAP1 targets were able to discriminate molecular class in primary tumors, so we performed unsupervised principal component analysis on the patient sample gene expression data using only genes identified by our analysis as BAP1 genomic targets. This identified a subset of 79 genes able to differentiate class 1 from class 2 tumors with clear discrimination of the tumors into two distinct groups (Fig. 5b). Enrichment analysis of these genes identified targets of ETS2, NF1, E2F4, and ELK1 in the discriminators that were upregulated in Class 2 tumors, and targets of FOXO4, CHX10, and SOX9 in the downregulated discriminators (see Additional file 10). These genes represent a direct link between the genomic function of BAP1 and the metastatic gene expression signature associated with BAP1 loss in UM tumors.

## Discussion

### Calling card methodology and chromatin modifying factors

The calling card methodology identifies genomic localizations of transcription factors that bind DNA directly [19]. We show that this novel approach can also identify genomic loci occupied by a chromatin-remodeling factor, BAP1, which does not bind to DNA directly. We found that fusions of BAP1 with transposase at either the N-terminus and C-terminus did not impair transposase function, and that while a substantial number (32%) of the genes were identified by both the N-terminal and C-terminal fusion constructs, the majority were identified by only one (Fig. 2b). By combining the lists of results from the two fusions, we generated a substantially larger number of statistically significant genomic loci, which may provide an important insight for future calling card studies of chromatin-remodeling factors.

### Genomic recruitment of BAP1 to regulate metastasis and differentiation

Upregulated BAP1-sensitive genes were associated with motif enrichment for Ets factors including ELF1, which has been shown previously in other cell types to co-precipitates with BAP1 [4]. Motifs found in both up-regulated and down-regulated data sets included NRF1 (nuclear respiratory factor 1) and YY1 (yin yang factor 1). NRF1 regulates metabolism, proteasome degradation, and mitochondrial biogenesis [29], and like ELF1, NRF1 has been shown previously in other cell types to co-precipitates with BAP1 [4], YY1 also co-precipitates with BAP1 [4], and YY1 regulates development, cell cycling, tumorigenesis, cell death, and a number of other pathways [30]. We suggest that recruitment of BAP1 by NRF1 and YY1 to specific genes requires the co-recruitment of tissue and lineage specific transcription factors to drive the aggressive nature of class 2 UM tumors.

We found pathways related to Epithelial-to-Mesenchymal Transition (EMT) and embryonic stem cell differentiation affected by depletion of BAP1 in UM cells. Prior studies of gene expression in response to loss of BAP1 have been performed in two other cell types, which showed substantial differences from each other, suggesting that the targets of BAP1 vary depending on cell lineage. In melanocytic cells, BAP1 is important for expression of genes involved in melanoblast and neural crest differentiation [10]. In contrast, in hematopoietic cells, loss of BAP1 affected pathways related to hematopoietic differentiation [31]. Although 45% of these genes were also targets of BAP1 binding in UM cells (35% of calling card genes were in the ChIP-Seq list), none of the hematopoietic differentiation genes responded to BAP1 depletion in our UM cells. In human non-small cell lung carcinoma cells (H1299 cells) [32], BAP1 depletion negatively regulated the FoxK2-target genes *MCM3*, *CDC14a*, and *CDKN1B*. Although two of these (*MCM3* and *CDC14a*) were targets of BAP1 binding in UM cells, we found no significant response in gene expression to BAP1 depletion. Taken together, these observations suggest that BAP1’s role in differentiation depends on specific changes in gene expression defined by other lineage-specific regulators. This agrees with our observation that BAP1 is recruited by NRF1 and YY1 to both up- and down-regulated genes. Thus, the pathways regulated by BAP1 that link its loss to metastasis – as it is in UM tumors – also depend on the tissue and lineage contexts of the tumor.

### Novel BAP1 targets are sufficient to identify aggressive tumors

The genomic loci identified by the calling card analysis represent bona fide, biologically valid, physiologically relevant targets of BAP1. First, genes adjacent to genomic loci targeted by BAP1 show differential expression when BAP1 is depleted: 70% of genes identified by calling-card analysis showed BAP1-sensitive expression; and conversely, 45% of all BAP1-sensitive genes were adjacent to genomic loci targeted in the calling-card analysis. Second, the genomic insertions identified by calling-card analysis clustered as narrow peaks near gene promoters with motifs known to bind transcription factors that interact with BAP1. Third, and most important, the subset of BAP1-responsive, Calling Card genes identified here are able to discriminate aggressive, class 2 primary tumor samples from human patients.

Identification of class 2 tumors is vital for clinical prognosis of the individual patient [27], and loss of BAP1 expression is seen in > 90% of metastasizing, class 2 tumors [7]. Comparing our BAP1-responsive, calling card target genes with the published expression data from primary UM patient samples [6], we identified several BAP1 genomic targets that were able to discriminate between class 1 and class 2 UM tumors.

## Conclusions

We used the calling card methodology to identify the genomic regions occupied by the chromatin-remodeling factor, BAP1, despite its inability to bind directly to DNA. This both expands the possible uses of the calling card technique to other chromatin-remodeling factors – as well as transcriptional co-activators and co-repressors – and identifies for the first time the genomic targets of BAP1 in UM cells. Far from targeting broad regions of modified histones, we found that BAP1 interacts with the genome at narrow regions around transcription start sites and is targeted to these sites by a specific set of DNA motifs. We suggest that BAP1 is recruited by NRF1, YY1, and Ets factors to specific sets of genes that are co-regulated by other tissue and lineage determinants. Importantly, several BAP1 genomic targets were able to discriminate aggressive class 2 UM tumors, demonstrating the first functional link between the role of BAP1 in chromatin remodeling and the phenotypic switch to metastasizing tumors. Moreover, this link suggests that the class 2 gene expression profile may serve as a functional readout for loss of BAP1 activity in UM tumors. Understanding the roles of these class-discriminating BAP1 genomic targets in the broader context of tumor progression will provide new insights into the cellular mechanisms of UM metastasis.

## Additional files

Additional file 1: BAP1-PBase genomic peaks. List of genomic regions of transposon insertion from the BAP1-PBase construct giving location, number of insertion events (hops), significance, and nearest gene (Feature). (XLSX 636 kb)

Additional file 2: PBase-BAP1 genomic peaks. List of genomic regions of transposon insertion from the PBase-BAP1 construct giving location, number of insertion events (hops), significance, and nearest gene (Feature). (XLSX 619 kb)

Additional file 3: Immunoblot to confirm knockdown of BAP1. Immunoblot of protein collected from the three independent experiments that were used for RNA-seq expressing control or BAP1-specific shRNAs in OCM-1A cells. Specific bands for BAP1 and GAPDH are indicated. (PDF 278 kb)

Additional file 4: Table summarizing the RNA-seq results. Differential gene expression results in BAP1-knockdown compared to control OCM-1A cells are shown from the RNA-seq data. Each row gives the unique Ensembl identifier, gene name, and description for each gene, as well as the log of the fold change (logFC), average expression, adjusted *p*-value, and linear fold change. (XLSX 1392 kb)

Additional file 5: BAP1-sensitive calling-card target genes. List of the 784 genes identified by the combination of > 2-fold BAP1-sensitive RNA expression changes and calling-card transposon targeting. Each row gives the gene name, unique Ensembl identifier, and description for each gene. Linear fold change and adjusted p-value in gene expression are from RNA-seq data. Experimental hops, p-value, and fusion construct data are from the calling card experiments. (XLSX 135 kb)

Additional file 6: Pathways associated with BAP1 target genes. The ten most significant pathways associated with BAP1 calling-card target genes that were up-regulated (positive response) or down-regulated (negative response) in response to BAP1 depletion. Pathways were identified by GSEA. (PDF 49 kb)

Additional file 7: GSEA terms associated with BAP1 target genes. Gene set enrichment analysis based on gene expression values for the 784 genes identified by the combination of > 2-fold BAP1-sensitive RNA expression changes and calling-card transposon targeting. Each row gives the name of the curated GSEA term, and the nominal enrichment score, *p*-value, false discovery rate (FDR), family-wise error rate (FWER), and rank-at-max value calculated by the GSEA software. (XLSX 23 kb)

Additional file 8: DNA motifs associated with BAP1 binding. HOMER was used to identify DNA motifs that were significantly enriched in genomic regions containing BAP1 calling card insertions. Calling card loci were divided into three categories based on response of associated genes to BAP1 depletion, and analyzed separately. (PDF 25 kb)

Additional file 9: Heatmap data. Gene expression values from published microarray data of primary UM tumors depicted in heatmap in Fig. 5a. Each row gives the gene rank assigned by comparative marker selection, which molecular class shows upregulation, and the name of each gene. Signal-to-noise ratio (SNR), *p*-values, false discovery rate (FDR), Bonferroni correction, family-wise error rate (FWER), fold change, means and standard deviations were all calculated by the comparative marker selection software. The original expression values from the published data [6] used to assemble the heatmap are included. (XLSX 196 kb)

Additional file 10: GSEA terms associated with top BAP1 target genes. Gene set enrichment analysis on the subset of 79 BAP1 targets that were able to discriminate molecular class in primary tumors. Each row gives the name of the curated GSEA term, the number of overlapping genes (size), enrichment score (ES), nominal enrichment score (NES), nominal p-value, false discovery rate (FDR), family-wise error rate (FWER), and rank-at-max value calculated by the GSEA software. (XLSX 12 kb)

## Abbreviations

GO: Gene ontology
GSEA: Gene set enrichment analysis
HOMER: Hypergeometric optimization of motif enrichment
PBase: *piggyBac* transposase
UM: Uveal melanoma
