# Supplementary figures and images for: Transposase mapping identifies the genomic targets of BAP1 in uveal melanoma
Source: BMC Med Genomics. 2018 Nov 6;11:97. doi: 10.1186/s12920-018-0424-0 (PMC6219186; doi:10.1186/s12920-018-0424-0)

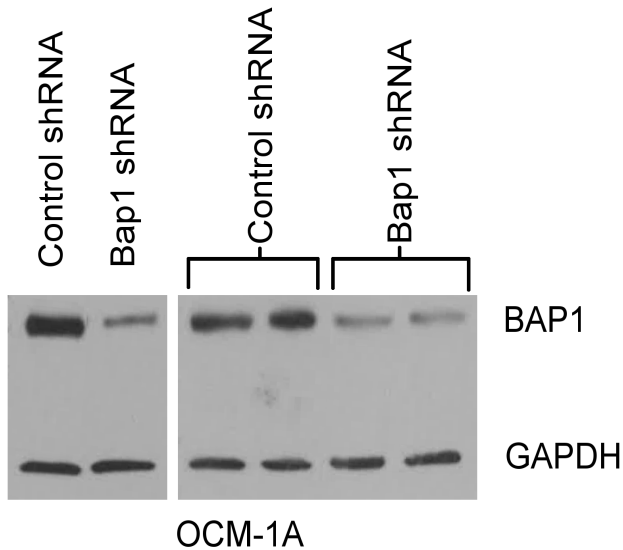

Supplement: Supplementary file 3 — Immunoblot to confirm knockdown of BAP1. Immunoblot of protein collected from the three independent experiments that were used for RNA-seq expressing control or BAP1-specific shRNAs in OCM-1A cells. Specific bands for BAP1 and GAPDH are indicated. (PDF 278 kb) [file 12920_2018_424_MOESM3_ESM.pdf]
