# Supplementary material for: Transposase mapping identifies the genomic targets of BAP1 in uveal melanoma
Source: BMC Med Genomics. 2018 Nov 6;11:97. doi: 10.1186/s12920-018-0424-0 (PMC6219186; doi:10.1186/s12920-018-0424-0)
Supplement: Supplementary file 6 — Pathways associated with BAP1 target genes. The ten most significant pathways associated with BAP1 calling-card target genes that were up-regulated (positive response) or down-regulated (negative response) in response to BAP1 depletion. Pathways were identified by GSEA. (PDF 49 kb) [file 12920_2018_424_MOESM6_ESM.pdf]

Table S6: Pathways associated with BAP1 target genes. The ten most significant pathways associated with BAP1 calling-card target genes that were up-regulated (positive response) or down-regulated (negative response) in response to BAP1 depletion. Pathways were identified by GSEA.

Pathways associated with positive response to BAP1-depletion

Description	p-value	FDR q-value
HIF-1-alpha transcription factor network	0.000000742	0.000218
Beta3 integrin cell surface interactions	0.00000186	0.00031
Direct p53 effectors	0.00000434	0.000444
ATF-2 transcription factor network	0.00000912	0.000713
Signaling events mediated by focal adhesion kinase	0.00000912	0.000713
ErbB1 downstream signaling	0.0000113	0.000774
BCR signaling pathway	0.0000147	0.00085
Regulation of retinoblastoma protein	0.0000147	0.00085
Beta1 integrin cell surface interactions	0.0000158	0.000878
BCR Signaling Pathway	0.0000283	0.00109

Pathways associated with negative response to BAP1-depletion

Description	p-value	FDR q-value
G alpha i Pathway	0.00000209	0.000155
PDGFR-beta signaling pathway	0.0000137	0.000728
EGF receptor (ErbB1) signaling pathway	0.0000385	0.0017
Mechanism of Gene Regulation by Peroxisome Proliferators via PPARa(alpha)	0.0000422	0.00178
Signaling events mediated by the Hedgehog family	0.0000894	0.00313
Beta1 integrin cell surface interactions	0.0000879	0.00313
Signaling events mediated by TCPTP	0.000106	0.00353
Direct p53 effectors	0.000144	0.00423
Myocyte Adrenergic Pathway is a specific case of the generalized Adrenergic Pathway.	0.000205	0.00569
Signaling events mediated by Hepatocyte Growth Factor Receptor (c-Met)	0.000256	0.00676
