# Supplementary material for: Transposase mapping identifies the genomic targets of BAP1 in uveal melanoma
Source: BMC Med Genomics. 2018 Nov 6;11:97. doi: 10.1186/s12920-018-0424-0 (PMC6219186; doi:10.1186/s12920-018-0424-0)
Supplement: Supplementary file 8 — DNA motifs associated with BAP1 binding. HOMER was used to identify DNA motifs that were significantly enriched in genomic regions containing BAP1 calling card insertions. Calling card loci were divided into three categories based on response of associated genes to BAP1 depletion, and analyzed separately. (PDF 25 kb) [file 12920_2018_424_MOESM8_ESM.pdf]

Table S8: DNA motifs associated with BAP1 binding. HOMER was used to identify DNA motifs that were significantly enriched in genomic regions containing BAP1 calling card insertions. Calling card loci were divided into three categories based on response of associated genes to BAP1 depletion, and analyzed separately.

Motifs associated with positive response to BAP1-depletion

Motif Name	Consensus Sequence	P-value	% of Target Sequences with Motif	% of Background Sequences with Motif
NRF1 (NRF)	CTGCGCATGCGC	0.0001	21.47%	10.25%
ETS (ETS)	AACCGGAAGT	0.0001	53.99%	39.06%
Unknown (Homeobox)	SSCMATWAAA	0.01	91.41%	83.53%
ELF1 (ETS)	AVCCGGAAGT	0.01	65.64%	54.73%
IRF1 (IRF)	GAAAGTGAAAGT	0.01	39.26%	29.01%
Elk4 (ETS)	NRYTTCCGGY	0.01	66.87%	56.24%
LXRE (NR)	RGGTTACTANAGGTCA	0.01	58.28%	47.49%
GABPA (ETS)	RACCGGAAGT	0.01	87.73%	79.83%
Mouse Recombination Hotspot (Zf)	ACTYKNATTCGTGNTACTTC	0.01	22.09%	14.93%

Motifs associated with negative response to BAP1-depletion

Motif Name	Consensus	P-value	% of Target Sequences with Motif	% of Background Sequences with Motif
NRF1 (NRF)	CTGCGCATGCGC	0.00001	22.73%	10.86%
ETS (ETS)	AACCGGAAGT	0.0001	53.98%	39.51%
GFX (E2F)	ATTCTCGCGAGA	0.001	5.68%	1.54%
E2F4 (E2F)	GGCGGGAAAH	0.001	56.82%	44.09%
Elk4 (ETS)	NRYTTCCGGY	0.001	68.75%	56.69%
NRF (NRF)	STGCGCATGCGC	0.001	23.86%	14.74%
Sp1 (Zf)	GGCCCCGCCCCC	0.01	48.30%	37.09%
Ronin (THAP)	RACTACAACTCCCAGVAKGC	0.01	6.82%	2.86%

Motifs associated with no response to BAP1-depletion

Motif Name	Consensus	P-value	% of Target Sequences with Motif	% of Background Sequences with Motif
ETS (ETS)	AACCGGAAGT	1E-12	57.51%	38.96%
ELF1 (ETS)	AVCCGGAAGT	1E-09	69.69%	54.48%
NRF1 (NRF)	CTGCGCATGCGC	1E-08	20.73%	10.69%
Elk4 (ETS)	NRYTTCCGGY	1E-07	70.47%	56.68%
E2F4 (E2F)	GGCGGGAAAH	1E-07	57.25%	43.54%
Elk1 (ETS)	HACTTCCGGY	1E-07	69.69%	56.65%
YY1 (Zf)	CAAGATGGCGGC	0.0001	18.13%	10.97%
Sp1 (Zf)	GGCCCCGCCCCC	0.0001	47.41%	37.05%
BMYB (HTH)	NHAACBGYYV	0.0001	99.48%	96.03%
GFX (E2F)	ATTCTCGCGAGA	0.0001	4.92%	1.68%
